# Deciphering the Resistance Mechanism of Tomato Plants Against Whitefly-Mediated Tomato Curly Stunt Virus Infection through Ultra-High-Performance Liquid Chromatography Coupled to Mass Spectrometry (UHPLC-MS)-Based Metabolomics Approaches

**DOI:** 10.3390/metabo9040060

**Published:** 2019-03-28

**Authors:** Leandri T. Rossouw, Ntakadzeni E. Madala, Fidele Tugizimana, Paul A. Steenkamp, Lindy L. Esterhuizen, Ian A. Dubery

**Affiliations:** Centre for Plant Metabolomics Research, Department of Biochemistry, University of Johannesburg, Auckland Park 2006, South Africa; leelybear@gmail.com (L.T.R.); ntaka.madala@univen.ac.za (N.E.M.); fideletu@gmail.com (F.T.); psteenkamp@uj.ac.za (P.A.S.)

**Keywords:** *tomato curly stunt virus*, defence, hydroxycinnamic acids, metabolomics, resistance tomato

## Abstract

Begomoviruses, such as the *Tomato curly stunt virus* (ToCSV), pose serious economic consequences due to severe crop losses. Therefore, the development and screening of possible resistance markers is imperative. While some tomato cultivars exhibit differential resistance to different begomovirus species, in most cases, the mechanism of resistance is not fully understood. In this study, the response of two near-isogenic lines of tomato (*Solanum lycopersicum*), differing in resistance against whitefly-mediated ToCSV infection were investigated using untargeted ultra-high-performance liquid chromatography coupled to mass spectrometry (UHPLC-MS)-based metabolomics. The responses of the two lines were deciphered using multivariate statistics models. Principal component analysis (PCA) scores plots from various time intervals revealed that the resistant line responded more rapidly with changes to the metabolome than the susceptible counterpart. Moreover, the metabolic reprogramming of chemically diverse metabolites that span a range of metabolic pathways was associated with the defence response. Biomarkers primarily included hydroxycinnamic acids conjugated to quinic acid, galactaric acid, and glucose. Minor constituents included benzenoids, flavonoids, and steroidal glycoalkaloids. Interestingly, when reduced to the level of metabolites, the phytochemistry of the infected plants’ responses was very similar. However, the resistant phenotype was strongly associated with the hydroxycinnamic acid derivatives deployed in response to infection. In addition, the resistant line was able to mount a stronger and quicker response.

## 1. Introduction

The tomato (*Solanum lycopersicum*) is one of the world’s most valuable agricultural commodities, accounting for approximately 14% of the worlds’ annual vegetable production [1]. This fruit is an important dietary source, which contains numerous health-beneficial bioactive phytochemicals, and active breeding programmes constantly produce new cultivars that possess superior and desirable traits [2]. However, due to a relatively narrow genetic base created by plant breeding selection, some pathogens can severely affect the yield and quality of tomato crops, creating a great need for methods to control them [3,4].

Viruses from the Begomovirus genus (family: Geminiviridea) are an important group of pathogens that infect tomatoes; Begomovirus is currently the largest genus in viral taxonomy (with 322 accepted species) [5]. A Begomovirus infection of a tomato often causes devastating symptoms, such as leaf chlorosis, leaf cupping, stunted growth, reduced fruit set, and, depending on the cultivar and time of infection, can lead to crop losses of up to 100% [6,7,8,9]. Begomoviruses are transmitted by the whitefly vector *Bemisia tabaci* [7]. *Bemisia tabaci* is considered to be a cryptic species complex, whose members display genetic, biological, and behavioural variation, but are morphologically indistinguishable [10,11]. Some members of this species complex have invaded well beyond their respective host ranges and cause devastating damage to crops. They cause damage directly through the feeding and contamination of plant products, as well as indirectly by acting as a vector for pathogenic plant viruses, particularly begomoviruses [12,13,14]. Over the last four decades, begomoviruses have emerged worldwide following the spread of their insect vector and have become one of the major constraints to open field tomato production [6,8,15].

Like most plant infecting viruses, begomoviruses represent an especially difficult group of plant pathogens to control. The most effective method, which is also the best economically and environmentally, appears to be breeding for resistance [15,16]. Wildtype tomato cultivars are a very good source of disease resistance, as many of these species do not succumb to begomovirus infection. Using this gene introgression approach in integrated breeding programs, new tomato cultivars with desirable characteristics, as well as resistance to infection by begomoviruses, have been developed [2,15,17,18,19,20,21].

The defence mechanisms involved in the resistance of crops to begomoviruses, have been extensively studied at the genome, transcriptome and proteome levels, but few have explored the metabolic alterations that occur in the host plant upon begomovirus infection [22]. Metabolic changes are the end-result of adaptive and defensive biochemical responses that take place upon infection. Because plants encounter many stimuli and stressors in their environments, they have developed mechanisms to deal with these factors, such as the ability to constitutively and actively synthesise thousands of low molecular weight secondary metabolites, which are essential for plant survival and defence [23,24,25]. The role of phytoanticipins and phytoalexins in plant innate immunity has previously been studied and it was found that the success or failure of an immune response depends on the speed and intensity with which it is mounted [26].

Metabolomic studies have previously been used investigate the general metabolism or biochemical responses of *Solanum* species to different stresses or phytopathogen infestations. These analyses include studies into organ-specific metabolites [20,27], salt stress response [28], metabolic alterations due to pathogen attacks, the susceptibility or resistance response to pathogens, and the beneficial interactions with mycorrhizal fungi [29,30,31,32,33]. Several metabolic studies have focused on the identification of metabolic pathways and metabolites that are perturbed by the infection of tomatoes by viruses, such as the *Tomato mosaic virus* (ToMV) [34]; *Tomato yellow leaf curl virus* (TYLCV) [22,35]; *Potato spindle tuber viroid* (PSTVd) [36], herbivore feeding [37], or by infestation with insects, such as spider mites (*Tetranychus urticae*) and aphids (*Myzus persicae*) [38,39]. All of these approaches have provided a deeper insight into biological processes and supported the discovery of potential biomarkers [40].

Here, the metabolic response of a begomovirus-resistant tomato line (**RT**) and its nearly isogenic susceptible counterpart (**S**), was investigated by comparing the production of defence-related secondary metabolites in response, firstly, to whitefly feeding alone (mock-inoculation), and secondly, to whitefly-mediated inoculation of a severe variant of *Tomato curly stunt virus* (ToCSV) [41].

## 2. Results

Upon whitefly-mediated inoculation of ToCSV, the **S** line (susceptible) developed symptoms typical of ToCSV infection (upper leaf yellowing, reduction in leaflet area, upward curling margins, and moderate to severely stunted internodes) compared to non-inoculated controls. The **RT** line remained symptomless and continued to develop normally, with flowering and fruit setting appearing similar to non-inoculated controls. Dot blot analysis (Figure S1.2 in Supplementary Materials) indicated that an antiviral host defence mechanism operates in the **RT** line, limiting viral accumulation and spread within the plant.

Metabolic changes occurring within tomato plants following infection with ToCSV were profiled by taking leaf samples from **RT** and **S** plants that were subjected to one of three different treatments: **Con** (control), **WF** (whitefly mock inoculation) or **WF + Vir** (whitefly-mediated inoculation of ToCSV). With day 1 being the first day of treatment, samples were collected on days 8, 15, 25 and 35 post-treatment. Aqueous methanol extracts of the samples were subjected to ultra-high-performance liquid chromatography coupled to mass spectrometry (UHPLC-MS) and the acquired data were analysed using multivariate data analysis (MVDA) modeling. With the aid of SIMCA (soft independent analysis of class analogy)-P + 13 software, principal component analysis (PCA) scores plots for each day were constructed.

**Figure 1:**
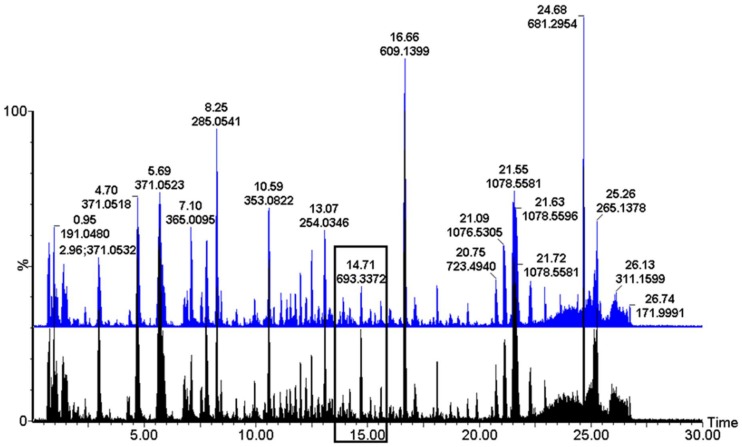
Overlay of representative original chromatograms obtained by ultra-high-performance liquid chromatography coupled to mass spectrometry (UHPLC-MS) of aqueous methanol extracts from tomato leaf tissue of viruliferous whitefly (**WF + Vir**) treated tomato plants of begomovirus-resistant tomato line **(RT)** (black) and its nearly isogenic susceptible counterpart (**S)** (blue) on day 8. MS chromatograms (*m*/*z* 100–1100) were acquired in ESI-negative mode. Retention times (in min) and accurate masses of the most intense signals are indicated in the chromatograms (plotted as base peak intensities (BPI), from 0 to 30 min). Some intensity differences (e.g., the boxed area) indicate comparative quantitative differences.

A visual comparison of UHPLC-MS chromatographic profiles of extracts of plants subjected to different treatments (Figure 1, Figure S2.1A/B) revealed no obvious qualitative differences between samples of different treatments (or different lines, **RT** vs. **S**, when the **WF + Vir** samples are compared as in Figure 1). This comparison does, however, reveal distinguishable quantitative differences of metabolites between differently treated samples. An example of these quantitative differences can be seen in the black boxes indicated in Figure 1, Figure S2.1A/B, which highlight the difference in intensity of a metabolite with *m*/*z* = 693.349 at Rt = 14.70 min between the differently treated samples.

**Figure 2:**
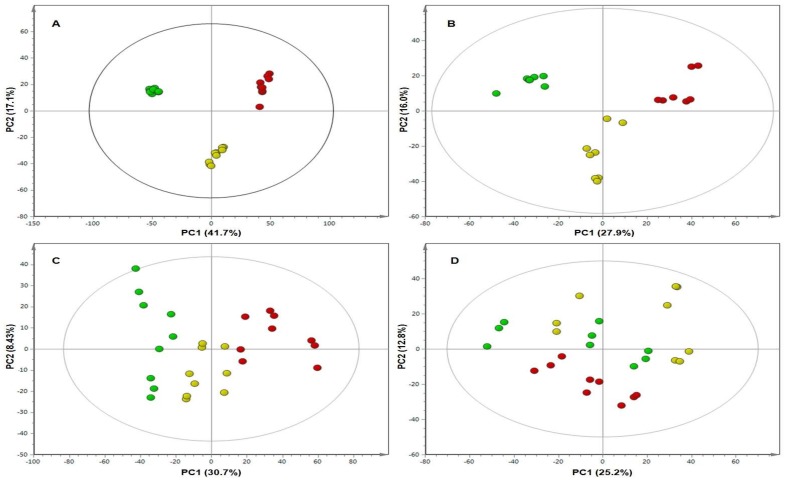
Principal component analysis (PCA) scores plots of negative ionisation data for the resistant (**RT**), tomato plants showing the differential clustering of the three different treatment groups at (**A**) day 8, (**B**) day 15, (**C**) day 25 and (**D**) day 35. Key: **Con**, (green); **WF**, (yellow); **WF + Vir** (red). PC, principal components.

**Figure 3:**
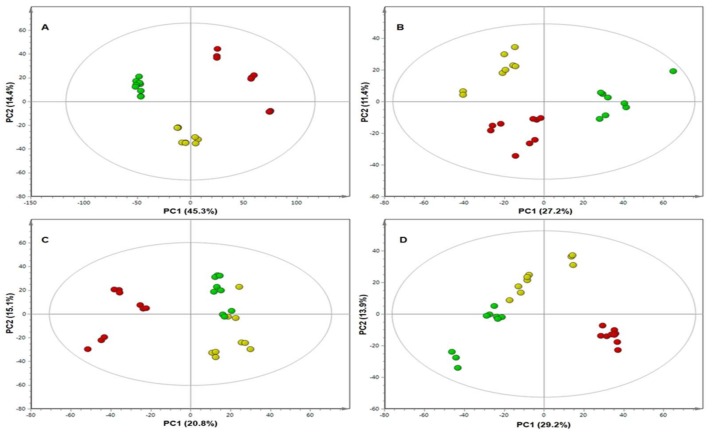
Principal component analysis (PCA) scores plots of negative ionisation data for the susceptible **(S)**, tomato plants showing the differential clustering of all three different treatment groups at (**A**) day 8, (**B**) day 15, (**C**) day 25 and (**D**) day 35. Key: **Con**, (green); **WF**, (yellow); **WF + Vir** (red). PC, principal components.

The metabolite profiles were compared and inspected in greater detail using MVDA models, specifically PCA scores plots and loading plots. PCA is an unsupervised multivariate linear model and often used for high-dimensional and complex metabolomic data analysis. PCA is a projection-based method and mathematically rigorous process that provides a global and qualitative visual representation of similarity and dissimilarity between and within samples (without using class information; e.g., treatment vs. control). The PCA scores plots of the different treatments on days 8, 15, 25, and 35 of each line revealed a remarkable differential pattern in the responses of the plants, as seen in Figure 2 and Figure 3.

**Figure 4:**
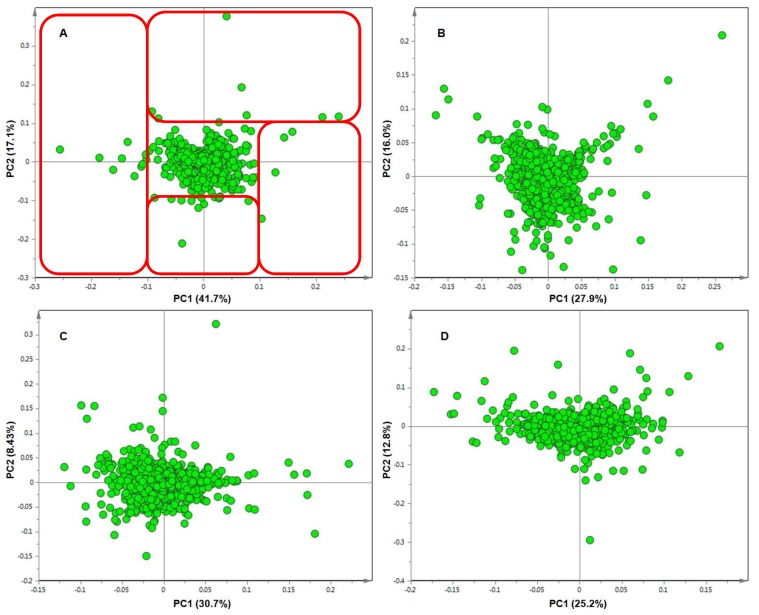
Principal component analysis (PCA) loadings plots of negative ionisation data for the resistant (**RT**) tomato plants. Shown are the metabolites (or biomarkers) responsible for the differential clustering (highlighted in the red boxes) seen on the scores plots of the different treatment groups at (**A**) day 8, (**B**) day 15, (**C**) day 25 and (**D**) day 35.

**Figure 5:**
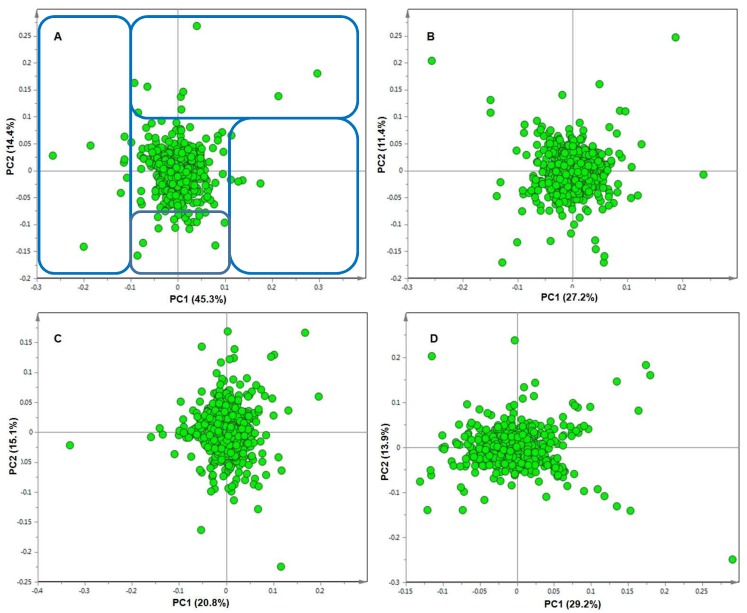
Principal component analysis (PCA) loadings plots of negative ionisation data for the susceptible (**S**) tomato plants. Shown are the metabolites (or biomarkers) responsible for the differential clustering (highlighted in the blue boxes) seen on the scores plots of the different treatment groups at (**A**) day 8, (**B**) day 15, (**C**) day 25 and (**D**) day 35.

As mentioned, the individual UHPLC-MS chromatograms (Figure 1, Figure S2.1A/B), displayed primarily qualitative differences between differently treated plants or between identically treated **RT** and **S** plants. PCA loadings plots (Figure 4 and Figure 5) did however, reveal which biomarkers were perturbed (up- or down-regulated), and by which treatments. These metabolites are listed, and where possible, annotated/identified, in Table 1. To decipher the separation seen on the PCA scores plots, the corresponding loading plots were evaluated to identify the metabolites responsible for the separation. Metabolites lying outside of 0.1 and −0.1 on both the x and y axes, as indicated by the red boxes (**RT**—Figure 4), or blue boxes (**S**—Figure 5), and tabulated in Table 1, were further analysed.

The ‘outliers’, representing metabolites of which the levels were perturbed by the treatment are presented in Table 1. The table also indicates in which treatment group, line, and on what day a specific metabolite was found to be perturbed. The association of these biomarkers with a specific treatment was further confirmed by the box-and-whiskers plots generated using the gas/liquid chromatography-mass spectrometry (XCMS) online software (File S2 in Supplementary Materials).

## 3. Discussion

The scores plots of **RT** on day 8, seen in Figure 2A, and that of **S** on day 8, seen in Figure 3A, show clear tight clusters of the individual samples (*n* = 3 biological repeats × 3 technical repeats) of each treatment group. The first principal components (PC1) of the scores plots of both the **RT** and the **S** lines are high (41.7% and 45.3% respectively), indicating large variance between the clustering groups. It is observed that the clustering of the **WF + Vir** group of **S** is slightly loose. This might be attributed to varying levels of defence activation in the susceptible viruliferous whitefly treated plants or lack of activation of a concerted defensive strategy at this point in time.

Despite the loose clustering of this treatment group, the three treatment groups still form clear clusters. The tight clustering of the differentially treated sample groups observed on the scores plot of **RT** and the clear clusters on the scores plot of **S** on day 8 indicate that the plants on day 8 mounted unique responses to their different treatments. We postulate that the clustering of the **Con** group on the left, the **WF** group in the middle, and the **WF + Vir** group on the right, indicate that the metabolic events of the **WF** group have similar metabolic elements to both the **Con** and the **WF + Vir** groups. However, the **WF** group is different from the control group, as the whitefly feeding on its own elicited a specific metabolic response, that is also different from the viruliferous whitefly feeding, as it lacks the metabolic response induced by the ToCSV infection.

The scores plots of **RT** and of **S** on day 15 (Figure 2B and Figure 3B, respectively) further highlight the clear differential clustering, but the clusters are seen to be less tightly grouped than those on day 8 for both **RT** and **S** (Figure 2A and Figure 3A respectively). The PC1 scores for **RT** and **S** (27.9% and 27.2% respectively) also decreased substantially from that of the day 8 PC1 scores, reflecting the decreasing variance between the different treatment groups on day 15. 

The positions of the different groups on the **S** scores plot on day 15 had changed from day 8, with the **WF** and the **WF + Vir** groups located to the left of the centre and the **Con** group alone on the right. These changes indicate that on day 15, the **WF** group and the **WF + Vir** group plants were metabolically more similar to each other than they were to the control group.

The scores plot of **RT** on day 25 (Figure 2C) shows extremely loose grouping of the different treatment groups. All three groups have moved closer together and the boundaries between them have become much less pronounced but are still present. The scores plot of **S** on day 25 (Figure 3C) shows that the clustering of the different treatment groups is approximately the same as that on day 15, with the clusters being more loosely grouped but still maintaining distinct groups. The PC1 score for **RT** (30.7%) is found to have increased slightly on day 15 but is still lower than that on day 8. The PC1 score of **S**, however, decreased (to 20.8%) in comparison to that on day 15. It was also observed on the **S** scores plot that the **WF** group moved closer to the **Con** group, and both were located on the right of the plot, while the **WF + Vir** group was found alone on the left. This indicates that the **WF** group was, on day 25, metabolically more similar to the **Con** group than to the **WF + Vir** group. These changes on day 25 indicate that the groups had become metabolically more similar, regaining a state of equilibrium. After the initial response induced by the whitefly feeding and the combination of whitefly feeding and virus inoculation, the **WF** treated plants and the **WF + Vir** treated plants (in the case of **RT**) returned to a neutral/basal state, i.e., regained homeostasis.

The scores plot of **RT** on day 35 (Figure 2D) shows a dramatic decrease in clustering compared to previous days; the **WF** and **Con** groups formed a single group and the **WF + Vir** group moved very close to the other two groups but retained its boundary. In contrast, it is observed from the scores plot of **S** on day 35 (Figure 3D) that the tightness of the clustering of the different treatment groups decreased slightly for the **Con** and **WF** groups from that of day 25, but the tightness of the **WF + Vir** group increased slightly. Distinct groups with clear boundaries are still seen.

The PC1 score for **RT** (25.2%) decreased slightly from that on day 25. These changes indicate that the **RT WF** group seemed to have completely regained homeostasis by day 35, while the **RT WF + Vir** group seemed to begin regaining homeostasis at day 35 but had not quite reached it. The PC1 score of **S** (29.2%) increased in comparison with that of day 25. Thus, the **S WF** group was still approaching homeostasis while the **S WF + Vir** group remained metabolically very different.

Ultimately, the PC1 scores were found to decrease from day 8 to day 35 for both **RT** and **S**, reflecting the decrease in variance present between the different treatment groups as the host’s response to viral infection progressed. The PC1 score of **S** on day 35, however, increased from that of day 25, indicating an increase in variance between the **Con**/**WF** grouping and the **WF + Vir** group.

The positions of the different treatment group clusters on the scores plots of **RT** on day 8, day 15, and day 25 show evidence of a pattern, with the **Con** group on the left, the **WF** group in the middle, and the **WF + Vir** group on the right. This pattern is maintained from day 8 through to day 25. On day 35, however, the pattern is no longer observed. Overall, it is observed that the three treatment groups moved closer together as the experiment progressed, resulting in the boundaries between groups becoming much less pronounced. On day 35, the **Con** group and the **WF** group appeared to blend together, as there was no longer a distinct boundary between these groups. The **WF + Vir** group on day 35 moved very close to the other two groups but could still be considered separate from them, as its boundary, although not very pronounced, was still present.

The positions of the different treatment group clusters on the scores plot for **S** on day 8 were observed to be the same as the pattern/layout observed for **RT** from day 8 to day 25. On day 15, the positions of the clusters changed with the **WF** and the **WF + Vir** groups moving closer to each other and to the left of the plot. While on day 25, the positions changed again, with the **WF** group moving closer to the **Con** group on the right, where the boundaries of these groups appear to overlap slightly. Despite the overlap, with the exception of a single **WF** plant, the two groups do not mix. On day 35 the different groups appear to be in the same positions as on day 25 (except for swapping left and right). The **Con** and **WF** groups also appear to retain their boundaries, as on day 25.

From the metabolomics data, ToCSV infection appeared to largely affect metabolites of the phenylpropanoid pathway (Table 1). Metabolites found to be perturbed range from phenols, flavonoids, and hydroxycinnamic acid esters to quinic acid (chlorogenic acids, CGAs) and galactaric acid. CGAs have recently been shown to play a significant role in plant defence and resistance to certain pathogens and also certain herbivores [42,43]. However, in almost all studies, CGA levels are seen to increase in response to attack and during resistance responses. In the current study, CGA levels in the whitefly mock-inoculated plants were found to decrease significantly compared to the control plants, while virus-inoculated plants showed a slight, further decrease in CGA (File S2 in Supplementary Materials). The whitefly feeding, therefore, had a greater impact on the CGA levels of the plants than the viral infection alone. The decrease in CGA levels was very substantial at day 8, while at day 35 the differences in the levels had become much less pronounced. Although the plants do not seem to return to normal (i.e., control) levels of CGAs by day 35 of this study, the levels of some of the isomers did appear to come close to those of the controls, highlighting the effect of whitefly feeding on these metabolites. These results are reflected in the PCA scores plots, where the grouping of the data indicates that the resistant line mounted a definite response early on (Figure 2), while a similar grouping appeared very late, post treatment, in the susceptible line (Figure 3). Therefore, the grouping seen on these PCA scores plots could be positively associated with (though not limited to) the depletion of CGAs.

The decrease in several isomers of CGAs, as observed in this study, could indicate a strategy to utilise a large existing pool of CGAs to mount a stronger defence response against stress, such as whitefly feeding and ToCSV infection. However, other implications of this decrease should also be considered. For instance, multiple studies have associated CGAs in resistance against fungal infection, where a decrease in CGA content was associated with increased susceptibility of the host plant [44,45,46]. The exact effects of whitefly feeding on the CGA content of tomato plants and the ultimate fate of the CGAs warrants further investigation.

In this study, the use of viruliferous whiteflies to infect plants with ToCSV adds another dimension to the study, as well as another form of stress to the plant, which contributed synergistically to the stress imposed by the ToCSV infection. The response of the plant to whitefly feeding would be expected to be limited to the beginning of the study, as whitefly feeding was limited to day 5. The ToCSV infection, however, progressed to day 35, the last time point investigated.

As no qualitative differences were found from the different treatments (in each line, as well as between lines) coupled with the information gained from the PCA scores plots, it is possible that the resistance associated with the **RT** line relies heavily on a rapid response after attack. From the patterns seen on the PCA scores plots and evaluation of the corresponding loading plots, it was noted that both the **RT** and **S** lines mount similar responses (thus using the same class of compounds) against the viral infection. However, the same PCA scores plots also show the **RT** line to mount the response much quicker than the **S** line, an ability which gives the **RT** line an advantage to get rid of the invading virus before damage is caused.

## 4. Materials and Methods

### 4.1. Plant Material

Seeds from two inbred near-isogenic tomato lines were obtained from TomaTech Ltd. (Rehovot, Israel). One line, **906-4**, was susceptible to the begomovirus *Tomato yellow leaf curl virus* (TYLCV – closely related to ToCVS) [41,47] infection (hereafter **S**) and the other, **902**, was resistant to TYLCV infection (hereafter **RT**). Both lines were issued from a breeding program aimed at introgressing TYLCV resistance genes from *Solanum habrochaites* (accessions LA386 and LA1777) into domestic susceptible *S. lycopersicum*. The pedigree of the **RT** and **S** lines has previously been described [18]. Seeds from another susceptible tomato cultivar, Rooikhaki, were obtained from Sakata Vegenetics (Lanseria, South Africa). All seeds were pre-treated in order to control seed borne pathogens by surface sterilisation in a 2.45% bleach (sodium hypochlorite) solution for 20 min. Subsequently, the seeds were rinsed in distilled water and incubated in a water bath at 30 °C for 25 min and a 50 °C bath for 10 min. Finally, the seeds were placed in a 10% tri-sodium phosphate buffer pH 8 for 15 min and allowed to dry. Further experimental details regarding *B. tabaci* whitefly colonies, viral culture, preparation of ToCSV infected plant material as source for virus acquisition and whitefly-mediated viral infection are described in File S1 in Supplementary Materials.

### 4.2. Experimental Design and Plant Treatments

Two identical, separate trials were conducted. For each trial, three identical seedling trays per cultivar (**RT** and S), were subjected to three different treatments, designated **Con, WF** and **WF + Vir.**

- **Con** (no treatment): a seedling tray, with 18 d old **S** and **RT** seedlings, received no whitefly or viral treatment and served as a control.
- **WF** (mock-inoculation): identical seedling tray of **S** and **RT** seedlings were placed into a cage. Whiteflies, which had been reared on healthy (i.e., virus-free) cotton and tomato plants (and had not been allowed to acquire the virus), were transferred onto the seedlings. These non-viruliferous whiteflies were allowed to feed on seedlings for four days.
- **WF + Vir** (whitefly-mediated ToCSV infection): in order to distinguish the metabolic response caused by viral infection and that caused by whitefly feeding alone, another identical seedling tray of 18 d old **S** and **RT** seedlings was placed into a different cage and the viruliferous whitefly transferred from a ToCSV infected cultivar (Rooikhaki) to the seedlings, where after the infected plant material was removed. The whiteflies were then allowed a four-day inoculation access period (IAP) on the seedlings. After four days, the whiteflies were removed from the seedlings, by shaking, and all the seedling trays, **Con**, **WF**, and **WF + Vir** moved to another room, where they were subjected to contact and systemic insecticide.

The sampling of leaf tissues was conducted on days 8, 15, 25, and 35 post treatment/infection. Nine plants were sampled per line (or treatment group) on each day, and three plant samples were pooled to create three biological replicates (A schematic diagram summarizing the experimental design is presented in Figure S1.1 in the Supplementary Materials).

### 4.3. Plant Material Collection

For both the **RT** and **S** lines, **WF** treated plants and **WF + Vir** treated plants were placed into cages with non-viruliferous whiteflies and viruliferous whiteflies respectively on day 1 and sampled on days 8, 15, 25, and 35. A single plant was never sampled twice throughout the experiment, in order to avoid biasness of the results by metabolites introduced during the wounding response during earlier sampling. On each of the days (days 8, 15, 25, and 35), nine individual plants per line were sampled from each of the three treatment groups (i.e., a total of 18 plants (9 **RT** + 9 **S**) from each of the treatment groups **Con**, **WF**, and **WF + Vir**). For each treatment and line, three biological repeats were each analysed for their metabolite content. Each biological replicate consisted of plant material pooled from three individual plants of the same line and treatment group, i.e., leaf plant material (2 g) from three plants per treatment were pooled (to make a total of 6 g), so that a total of three samples (biological replicates) per treatment were analysed for each line. This process was done on each sampling day, except on day 8, where the plants were too small to harvest 2 g from each, resulting in day 8 samples being made up of 1 g of plant material from each of the pooled plants, totaling 3 g per sample per treatment and line. Throughout the sampling trials, the leaf samples were collected and immediately snap frozen in liquid nitrogen to quench metabolic activity.

### 4.4. Metabolite Extraction

Three (3) g of frozen leaf samples (ground in a mortar and pestle) were mixed with 30 mL of 80% analytical grade aqueous methanol and homogenised using an ultraturrax rotating blade homogeniser. The homogenates were placed in a warm bath at 50 °C for 15 min and centrifuged at 5100× *g* for 15 min. After centrifugation, the supernatants were poured into a clean round bottom flask and concentrated to approximately 2 mL using a rotary evaporator at 50 °C. The remaining 2 mL of each sample was further evaporated to dryness using a centrifugal evaporator at 50 °C. Samples were then reconstituted in 0.5 mL of 50% high purity aqueous methanol. Reconstituted samples were filtered through 0.22 µm nylon filters into pre-slitted chromatography vials fitted with 300 µL inserts. These samples were tightly capped and stored at −20 °C until analysis by ultra-high-performance liquid chromatography coupled to electrospray ionisation mass spectrometry (UHPLC-ESI-MS).

### 4.5. UHPLC-ESI-MS Analysis

Aqueous methanol sample extracts were analysed on a Waters Acquity Classic UHPLC system coupled to a Waters ultra-high definition Synapt G1 MS instrument (Waters Corporation, Manchester, UK) equipped with an Acquity HSS T3 C18 column (150 × 2.1 mm with a particle size of 1.8 µm) (Waters Corporation, Manchester, UK). Each sample was analysed three times to eliminate any possible technical errors. The composition of mobile phase A consisted of 0.1% formic acid (*v*/*v*) in deionised water and mobile phase B consisted of 0.1% formic acid (*v*/*v*) in methanol. Each sample (1 μL) was analysed using a 30 min method comprising of the following gradient conditions: a constant flow rate of 0.4 mL/min. The mobile phase was initially made up of 95% A and 5% B and was kept that way for the first 5 min, after which solvent A was decreased to 45% and B was increased to 55% over the course of 17 min (i.e., up to 22 min). The composition of the mobile phase was then changed to 5% A and 95% B over the course of 3 min (22–25 min) and remained that way for a further minute (25–26 min). Over the course of the next minute (26–27 min) the mobile phase was returned to its initial composition (95% A and 5% B) and kept at the initial conditions for a further 3 min until the end of the run (27–30 min).

The Synapt G1 quadrupole time of flight (QTOF) high definition MS detector (Waters Corporation, Manchester, UK) was coupled in series after the photo-diode array (PDA) detector. MS data were acquired in both positive and negative electrospray ionisation (ESI) modes. For MS detection, the optimal experimental conditions were as follows: capillary voltage of 2.0 kV, sample cone voltage of 35 V, collision energy ramp start of 10 eV and end of 30 eV (to generate a typical MS^E^ data), source temperature of 120 °C, desolvation temperature of 400 °C, cone gas flow of 50 L/h and desolvation gas flow of 800 L/h. MS data were acquired in the *m*/*z* range of 100–1100 Da with a scan time of 0.100 sec and an inter-scan time of 0.020 sec. Collected data were in centroid format and mass spectra corrected in real time by an external reference standard. The external reference standard consisted of leucine-enkephalin (5 pg/mL) using a lock-mass sprayer interface and a constant lock-mass flow rate of 0.2 mL/ min.

Metabolite annotation indicated the high prevalence of phenolic acids and chlorogenic acids amongst the statistically significant biomarkers (Section 4.7). These metabolites ionise better in negative ionisation mode [48,49,50,51], and advanced MS^E^ experiments were, therefore, conducted in the negative ionisation mode. Here, the same basic settings were used except for the collision energy that was ramped from 10 to 60 eV.

### 4.6. Multivariate Data Analysis

For pre-processing and analysis of raw UHPLC-ESI-MS data, MassLynx XS™ software version 4.1 (Waters Corporation, Manchester, UK) with an advanced statistical program for multivariate data analysis (MVDA), MarkerLynx™, was used. Raw data was extracted and preprocessed using MarkerLynx^TM^ software. The MarkerLynx^TM^ method parameter settings for preprocessing of raw data were set as follows: retention time (Rt) 0.50–26 min with the Rt window as 0.20 min, mass range of 100–1200 Da, the mass tolerance as 0.05 Da and the intensity threshold set to 20 counts. Peak width at 5% height as well as peak-to-peak baseline noise were both set to be automatically calculated with applied smoothing.

The dataset obtained from MarkerLynx^TM^ preprocessing was exported to the SIMCA-P+ software version 13.0 (Umetrics, Umea, Sweden) program for principal component analysis (PCA) computation. All data was centroid and subsequently Pareto scaled using SIMCA-P+ 13.0. PCA scores plots, as well as loadings plots, were constructed using the same software. From the loading plots, corresponding to the PCA scores plots of each dataset, metabolites or biomarkers that had been significantly perturbed (those lying outside of 0.1 and –0.1 of both the x and y axes of the loadings plot) were selected. The *m*/*z* and Rt of the selected metabolites were noted. Using MassLynx XS™ the selected metabolites were located on sample chromatograms and their MS spectra viewed. From the MS spectra the elemental composition tool was used to generate molecular formulae of the pseudo-molecular ions ([M-H]^™^), and formulae with a mass difference, between the measured and calculated mass, of below 5 milliDalton (mDa) were selected.

### 4.7. Metabolite Annotation and Semi-Quantitation

The selected molecular formulae derived for the signatory biomarkers were used to search the Dictionary of Natural Products [52] and published literature [48,49,50,51,53] for possible compound identities. Signatory biomarkers were annotated to level 2 of the Metabolomics Standards Initiative (MSI) [54]. XCMS-online [55] was used to obtain box-and-whiskers plots of the annotated metabolites in order to compare their relative levels in differently treated plants. Data were uploaded and processed using the multi-group function on XCMS-online. The parameter method ‘UPLC/UHD Q-sTOF’ was used with polarity set to negative and the Rt correction method was set to peak groups (missing number of samples): 1, non-linear/linear alignment: loess (locally weighted smoothing) and extra (number of peaks): 1. Statistical parameters of the analyses remained unchanged, with a *p*-value threshold for highly significant features of 0.01 and a *p*-value threshold for significant features of 0.05. Features detected with a *p*-value > 0.05 were considered not significant.

## 5. Conclusions

This study represents a unique approach, where metabolomics were used, instead of the traditionally preferred proteomic and transcriptomic approaches, to investigate virus-plant interaction. Apart from the ability to identify possible defensive biomarkers (metabolites in this case), our study has also shown that metabolomics tools can further uncover the hidden intrinsic interplay between metabolites (as shown in this study with PCA scores plots) during the mounting of defence responses by plants against pathogens. As such, it can be concluded that metabolomic approaches add a new dimension for the identification and elucidation of the defensive capabilities and differences between resistant and susceptible lines.

Several metabolites, associated with different related metabolic pathways, were identified as potential biomarkers. Unfortunately, the chemical nature of some of the identified signatory ions remains unknown, but it is likely that the resistance response towards ToCSV is determined by a cocktail of defence-related metabolites, rather than a selected few from a specific subset of phytochemicals. Metabolomics insights into phenotypic traits, such as resistance suggest the importance of the regulation of a delicate balance of secondary metabolite combinations of diverse biosynthetic origins as opposed to the regulation of a small number of vital biomarkers [56]. In the case of near-isogenic lines, as used in this study, this insight is of even greater importance, since the relative abundance of the metabolites, either as phytoanticipins or as phytoalexins, is probably linked to each line’s capacity to preferentially utilize the metabolite resources at its disposal to resist and fend off the viral infection. In order to fully understand the mechanism of resistance in tomato plants, it is important that these biomarker compounds are identified and studied further to fully comprehend the extent of the metabolic perturbations caused by whitefly feeding and viral infection and the resulting host responses.

## Figures and Tables

**Table 1:** Probable identities (*m*/*z*, retention times and elemental composition) of the annotated metabolites/biomarkers lying outside the −0.1 to 0.1 range on both the x and the y axes of the loadings plots for **RT** (Figure 4) and **S** (Figure 5) of control (**Con**) versus whitefly (**WF**) and versus viruliferous whitefly (**WF + Vir**). The table also shows in which treatment group, of which line on which day each annotated metabolite was identified as a signatory biomarker.

*m*/*z* [M−H]^−^	Rt (Min)	Elemental Formula	Annotation/Identity	Treatment, Line (RT vs. S) and Day (8, 15, 25, 35) that Metabolite Was Identified as a Biomarker		
				Con	WF	Wf + Vir
163.0395	10.80	C_9_H_8_O_3_	Coumaric acid	#	#	RT-8
191.0556	10.59	C_7_H_12_O_6_	Quinic acid	RT-8, S-8	RT-8, S-8	#
209.0297	7.79	C_6_H_10_O_8_	Galactaric acid	RT-8	RT-8, S-8	#
221.0189	16.01	-	Unknown ^b^	#	RT-8, S-8	RT-8, S-8
245.0926	13.91	C_13_H_14_N_2_O_3_	Acetyl tryptophan	#	RT-8, S-8	S-8
249.1150	6.93	C_13_H_18_N_2_O_3_	Caffeoyl putrecine	#	RT-8	S-8
251.0867	17.13	-	Unknown ^b^	#	#	RT-8, S-8
254.0360	13.08	-	Unknown ^b^	RT-8	#	RT-8, S-8
270.0311	11.77	-	Unknown ^b^	S-8	#	RT-8, S-8
284.0532	8.25	C_12_H_13_O_8_	2,3-Dihydroxybenzoic-3-O-β-D-xyloside ^a^	#	RT-8, S-8	RT-8, S-8, S-35
291.1261	8.34	-	Unknown ^b^	#	S-35	#
291.1251	10.28	-	Unknown ^b^	#	S-35	S-35
307.1210	8.82	-	Unknown ^b^	#	S-35	S-35
321.1361	9.84	-	Unknown ^b^	#	S-35	S-35
351.1212	11.42	-	Unknown ^b^	#	#	RT-8, S-8, S-35
353.0873	7.89	C_16_H_18_O_9_	3-O-Caffeoylquinic acid ^a^	S-8	S-8, S-35	S-35
353.0873	10.58	C_16_H_18_O_9_	5-O-(E)-Caffeoylquinic acid ^a^	RT-8, S-8, S-35	RT-8, S-8, S-35	#
353.0873	10.58	C_16_H_18_O_9_	5-O-(E)-Caffeoylquinic acid ^a^	#	RT-8, S-8	#
353.0873	11.09	C_16_H_18_O_9_	4-O-Caffeoylquinic acid ^a^	RT-8, S-8, S-35	RT-8, S-8, S-35	#
353.0873	12.24	C_16_H_18_O_9_	5-O-(Z)-Caffeoylquinic acid ^a^	RT-8, S-8	#	#
355.1029	11.76	C_16_H_20_O_9_	Ferulic acid glycoside ^a^	S-35	#	S-35
365.0089	7.11	-	Unknown ^b^	#	RT-8, S-8	RT-8, S-8, S-35
367.1029	13.41	C_17_H_20_O_9_	5-O-Feruloylquinic acid ^a^	RT-8, S-8, S-35	S-8	#
371.0614	7.77	C_15_H_16_O_11_	5-O-Caffeoylgalactaric acid ^a^	RT-8, S-8, S-35	RT-8, S-8, S-35	#
385.1135	13.30	C_17_H_22_O_10_	Sinapoylglycoside ^a^	S-8, S-35	RT-8, S-8, S-35	#
401.1448	12.49	C_18_H_26_O_10_	Benzyl alcohol-hexose-pentose^a^	#	RT-8, S-8	RT-8, S-8, S-35
431.1849	13.30	-	Sinapoylglycoside FA ^a^	#	RT-8, S-8	S-8
447.1440	12.57	-	Benzyl alcohol-hexose-pentose FA ^a^	#	#	S-35
571.1299	8.38	C_24_H_28_O_16_	2,3-Dihydroxybenzoic-3-O-β-D-xyloside (dimer) ^a^	#	#	S-35
583.2713	10.27	-	Unknown (dimer of 291) ^b^	#	S-35	S-35
593.1472	18.10	-	Unknown	RT-8, S-8, S-35	#	#
609.1456	16.66	C_27_H_30_O_16_	Quercetin 3-rutinoside ^a^	RT-8, S-8, S-35	S-35	RT-8, S-8, S-35
615.2621	8.81	-	Unknown (dimer of 307) ^b^	#	S-35	S-35
675.1130	6.99	C_22_H_30_NO_23_	^b^	#	RT-8, S-8	RT-8
677.1507	19.49	C_34_H_30_O_15_	^b^	#	RT-8, S-8	RT-8, S-8
693.3487	14.7	C_37_H_50_N_4_O_9_	N^1^,N^4^,N^12^ tris (dihydroxycaffeoyl)spermine	#	RT-8	RT-8, S-8
707.1823	10.58	C_32_H_36_O_18_	5-O-(E)-Caffeoylquinic acid (dimer) ^a^	RT-8, S-8, S-35	RT-8, S-8	#
735.2136	13.53	C_34_H_40_O_18_	5-O-(E)-Feruloylquinic acid (dimer) ^a^	#	#	S-35
741.1878	15.59	C_32_H_38_O_20_	Quercetin-3-O-deoxyhexose-O-hexose-O-pentose ^a^	RT-8, S-8	S-35	#
743.1307	7.78	C_15_H_16_O_11_	5-O-(E)-Caffeoylgalactaric acid (dimer) ^a^	RT-8, S-8, S-35	RT-8, S-8	#
771.1984	12.76	C_33_H_40_O_21_	Quercetin-3-rutinoside-7-glycoside ^a^	S-35	#	#
947.2514	18.41	-	Unknown ^b^	S-35	S-35	#
1094.5383	19.09	C_51_H_85_NO_24_	Hydroxytomatine (Lycoperoside H)-FA	RT-8	RT-8, S-8, S-35	S-35

Supporting evidence for structural elucidation are supplied in Supplementary File S2. ^a^ Verified using MS/MS data; ^b^ Identities unknown despite having MS/MS data; FA= formic acid adduct; #=Specific metabolite not found to be perturbed in treatment group.

## Data Availability

The study design information (Figure S1 in Supplementary Materials), LC-MS raw data, analyses and data processing information, and the meta-data are being deposited to the EMBL-EBI metabolomics repository – MetaboLights50, with the identifier MTBLS895 (http://www.ebi.ac.uk/metabolights/MTBLS.895). In addition, UHPLC-MS chromatograms and MS/MS fragmentation spectra of signatory biomarker metabolites identified through multivariate data analysis are presented in File S2 in Supplementary Materials.

## Supplementary Materials

The following are available online at https://www.mdpi.com/2218-1989/9/4/60/s1. Supplementary File S1: Extended experimental procedures. Supplementary File S2: UHPLC-MS chromatograms and MS/MS fragmentation spectra of signatory biomarker metabolites identified through multivariate data analysis.
